# Deadliest Enemy: Our War against Killer Germs

**DOI:** 10.3201/eid2401.171081

**Published:** 2018-01

**Authors:** Amesh A. Adalja

**Affiliations:** Hopkins Center for Health Security, Baltimore, Maryland, USA

**Keywords:** outbreaks, policy, pandemics, germs, war, infectious diseases, viruses, bacteria

In the world of infectious diseases, there are many microorganisms that regularly infect humans. However, certain infectious diseases have special significance to society and persons therein.

In Michael T. Osterholm and Mark Olshaker’s book *Deadliest Enemy: Our War against Killer Germs *(Figure), the explicit purpose is to provide “a new paradigm for the threats posed by infectious disease outbreaks in the twenty-first century.” This fast-paced and attention-grabbing book is focused on “those maladies with the potential to disrupt the social, political, economic, emotional, or existential well-being of large regions, or even the entire planet.”

**Figure:**
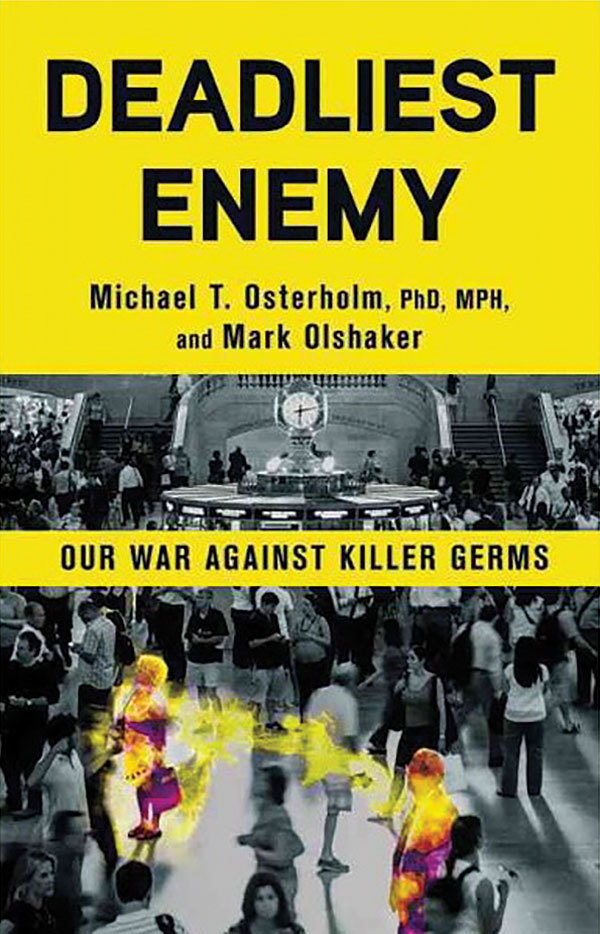
Michael T. Osterholm and Mark Olshaker’s book Deadliest Enemy: Our War against Killer Germs

To anyone within the field of infectious diseases, Osterholm is a familiar, well-regarded scientist who has provided guiding insight not only to the state of Minnesota, where he was state epidemiologist, but to the entire country and world. The main value of this book is that it is an overview and analysis informed by Osterholm’s unique expertise. Olshaker, his coauthor, is an extensively published writer and novelist of works such as *Mindhunter*.

The book contains 14 chapters, each addressing a major infectious disease issue. Beginning with HIV/AIDS, the book covers every major outbreak in the past 3 decades, including severe acute respiratory syndrome, Middle East respiratory syndrome, toxic shock syndrome, Zika virus disease, and Ebola. Osterholm’s personal encounter with La Crosse encephalitis is harrowing. In addition, all major policy issues in the field over the past 3 decades, including bioterrorism, gain-of-function influenza research, the antivaccine movement, and antimicrobial resistance, are covered in detail.

The book is also valuable because it goes beyond a mere journalistic description of diseases and their impact as it actively offers policy- and scientific-based approaches for addressing the problems described. In the chapter titled “Taking Influenza Off the Table,” the concept of “game-changing influenza vaccines” is introduced, providing a compelling rationale and strategy for developing vaccines in a realm where traditional business and technological approaches fall short. Similarly, “Fighting the Resistance” is focused on potential solutions to the public health emergency of antimicrobial drug resistance.

The most insightful aspect of the book is the authors’ threat matrix. In this clarifying matrix, 4 classes of threats are identified: pathogens of pandemic potential; pathogens of critical regional importance; bioterrorism, dual-use research of concern, and gain-of-function research of concern; and endemic diseases. After subdividing infectious diseases into this matrix, the authors offer an easily understood and highly accessible agenda with several priority items to support humans against these threats.

One of the underlying themes of this book, which follows some conventions of a mystery novel in how the authors structure the chapters, is the emphasis on solving puzzles of infectious diseases through first-hand, diligent, logical thinking integrated with proper technology and appropriate laboratory studies. A good example is when one has to “play public policy Jeopardy,” as Osterholm has with contentious issues involving gain-of-function research of concern.

Osterholm’s mention of his childhood love of Sherlock Holmes and other mysteries is the leitmotif of this excellent book. This engaging book, which is easily accessible and requires no technical knowledge, will appeal to a broad range of practitioners engaged in public health and clinical management of infectious diseases, biomedical trainees interested in infectious diseases, and policy makers.

